# Current therapeutic strategies and perspectives in refractory ITP: What have we learned recently?

**DOI:** 10.3389/fimmu.2022.953716

**Published:** 2022-08-08

**Authors:** Yue Lv, Huiping Shi, Hong Liu, Lu Zhou

**Affiliations:** ^1^ Department of Hematology, Affiliated Hospital and Medical School of Nantong University, Nantong, China; ^2^ Soochow University Medical College, Suzhou, China

**Keywords:** refractory immune thrombocytopenia, autoimmunity, platelet, fostamatinib, desialylation

## Abstract

Immune thrombocytopenia (ITP) is an acquired autoimmune bleeding disorder featured by increased platelet destruction and deficient megakaryocyte maturation. First-line treatments include corticosteroids, intravenous immunoglobulin and intravenous anti-D immunoglobulin. Second-line treatments consist of rituximab, thrombopoietin receptor agonists and splenectomy. Although most patients benefit from these treatments, an individualized treatment approach is warranted due to the large heterogeneity among ITP patients. In addition, ITP patients may relapse and there remains a subset of patients who become refractory to treatments. The management of these refractory patients is still a challenge. This review aims to summarize emerging therapeutic approaches for refractory ITP in several categories according to their different targets, including macrophages, platelets/megakaryocytes, T cells, B cells, and endothelial cells. Moreover, current management strategies and combination regimens of refractory ITP are also discussed.

## 1 Introduction

Immune thrombocytopenia (ITP) is an acquired autoimmune bleeding disorder featured by increased platelet destruction and deficient megakaryocyte maturation. The diagnostic threshold for ITP is a platelet count less than 100×10^9^/L (1). Over the past decade, the incidence of ITP is approximately 3 to 4 patients in every 10,000 individuals per year, with a slightly higher incidence in women (2). ITP patients display varying bleeding symptoms, ranging from mild petechiae, purpura, epistaxis to severe gastrointestinal or intracranial bleeding, which might be life-threatening. Depending on the duration of the disease, ITP is divided into three categories: newly diagnosed (less than 3 months), persistent (between 3 and 12 months), and chronic (more than 12 months) (3). For the clinical treatment effectiveness, the International Working Group (IWG) defines complete response (CR) as a platelet count of ≥100×10^9^/L without bleeding and response (R) as a platelet count of ≥ 30×10^9^/L with at least a 2-fold rise in baseline platelet count and no bleeding. In addition, the speed of response onset and duration are also essential in assessing efficacy.

Currently, the definition of refractory ITP is still controversial. “Refractory” was initially defined as failure or recurrence after splenectomy (1). In 2016, the definition expanded to include not only those splenectomy non-responders but also those intolerant or unwilling to undergo splenectomy (4). In another study, authors defined those not responding to splenectomy, rituximab, romiplostim and eltrombopag as refractory ITP patients (5). More recently, refractory ITP was defined as patients unresponsive to one or more single-agent therapies, without reference to splenectomy (6). About 10% of ITP patients show poor or no response to treatments and subsequently develop refractory ITP (7). These patients exhibit lower platelet counts, severe risk of bleeding and infection with a significantly lower life quality and higher mortality (8, 9). First-line and rescue treatments consist of steroids, intravenous immunoglobulin (IVIG) and intravenous anti-D immunoglobulin (anti-D). Second-line therapies consist of rituximab, thrombopoietin receptor agonists (TPO-RAs) and splenectomy. Additional second-line agents include immunosuppressants (e.g., azathioprine, mycophenolate mofetil, cyclosporine and others). With advent of these therapeutic options, a greater portion of patients achieve favorable clinical outcomes. Nonetheless, a subset of refractory ITP patients still presents as non-responders. Treatment for patients unresponsive to TPO-RAs and rituximab tends to be more difficult (6). There is an unmet need for more curative therapies, representing a significant challenge for clinicians.

It has been proved that combination therapy works better than single-agent therapy and targeting multiple biological mechanisms concurrently is more beneficial for those highly refractory ITP patients (6, 10). TPO-RA and rituximab have been painstakingly delineated in numerous reviews, so here we focus on other novel drugs. In this review, we summarize available agents with novel mechanisms of action according to different therapeutic targets including macrophages, platelets/megakaryocytes, T cells, B cells, and endothelial cells. Moreover, we discuss current management strategies and combination regimens of refractory ITP.

## 2 Pathogenesis of ITP

**Figure 1:**
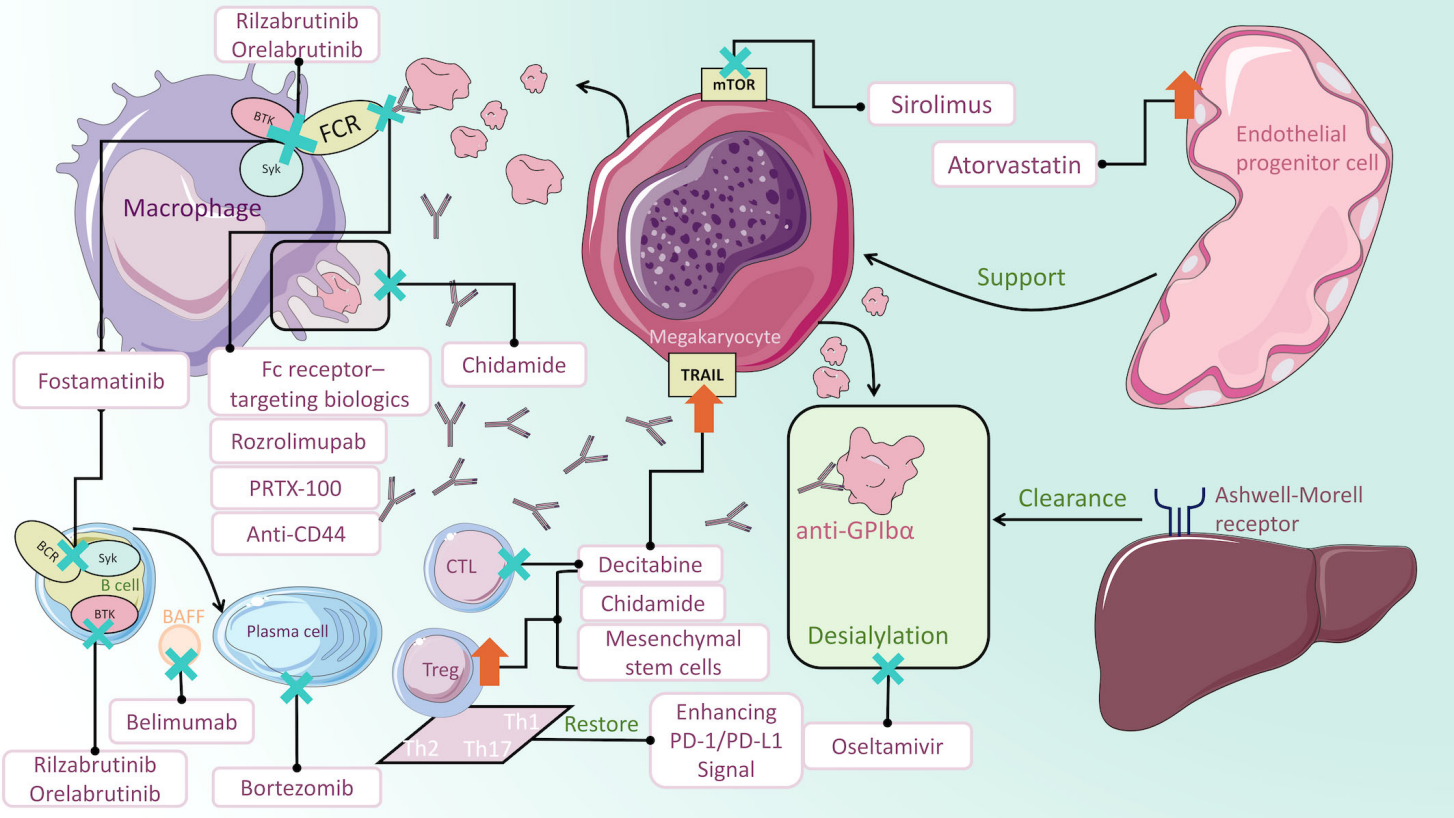
Mechanisms of ITP medications. Fostamatinib impairs Syk-mediated phagocytosis of platelets. Rilzabrutinib and orelabrutinib block FcγR signaling pathway transduction by inhibiting BTK. PRTX-100, Fc receptor-targeting biologics, rozrolimupab and anti-CD44 inhibit macrophage phagocytosis by blocking IgG binding to Fc receptors. Oseltamivir inhibits glycoprotein-induced desialylation, protecting platelets from clearance by hepatocyte Ashwell-Morell receptors. Sirolimus acts on mTOR on megakaryocytes to regulate autophagy. Decitabine not only promotes megakaryocyte maturation but also regulates Treg cells and CTLs. Enhancement of the PD-1/PD-L1 signaling pathway could restore the balance of Th cells. Chidamide increases the immunosuppressive functions of Treg cells and inhibits macrophage phagocytosis. Mesenchymal stem cells could upregulate Treg cells. Bortezomib stimulates apoptosis in LLPCs and short-lived plasma cells, thus decreasing antiplatelet antibodies. In the context of depleting B cells, adding Belimumab to block BAFF can reduce the number of splenic plasma cells. Atorvastatin improves endothelial progenitor cells function to promote megakaryopoietic production.

The pathogenesis of ITP is complex and not fully understood. Fcγ receptor (FcγR)-mediated macrophage phagocytosis causes enhanced platelet clearance. During the process, Spleen tyrosine kinase (Syk) and Bruton tyrosine kinase (BTK) are engaged in the FcγR signal transduction and have become novel treatment targets. Besides, Staphylococcal protein A (SpA) was found to inhibit macrophage phagocytosis by binding with the IgG Fc region (11). Similarly, recombinant therapeutics are developed to be alternative to IVIG and anti-D for the inhibition of antibody-coated platelet destruction (12, 13). Megakaryocytes produce platelets in the bone marrow, and their abnormality is also an essential cause of ITP. Platelet desialylation often leads to refractory ITP due to the completely different Fc-independent platelet clearance pathway. Anti-GPIbα antibodies induce desialylation of platelets, which are recognized by hepatocyte Ashwell-Morell receptors and cleared in the liver subsequently (14). Moreover, loss of autophagy could impede megakaryopoiesis and thrombopoiesis (15). Enhancing platelet autophagy could alleviate platelet destruction (16). Furthermore, DNA methylation participates in megakaryocyte maturation (17). T cells have also been implicated in the development of ITP. Dysfunctional T cell subsets are related to the loss of immunological tolerance. Cytotoxic T cells (CTLs) can directly lyse platelets. Activating programmed death (PD-1)/programmed death ligand-1 (PD-L1) signaling pathway could restore the balance of cytokines that are related to Th cells (18). DNA methylation and histone acetylation abnormality induced deficient Treg cells (19, 20). The former could also enhance the platelet-damaging effect of CTLs (21). Mesenchymal stem cells (MSCs) could regulate immune cell homeostasis, serving as therapeutic targets for refractory cases (22). Anti-platelet antibodies not only mediate platelet destruction but also affect thrombocytogenesis by inhibiting megakaryocyte production and maturation (23). B cells differentiate into plasma cells which persistently secrete anti-platelet antibodies. Previous mainstream therapies only suppress B cells or short-lived plasma cells rather than long-lived plasma cells (LLPCs) (24). Depleting LLPCs could be of interest in steroid-resistant or relapsed ITP. In the terms of endothelial function, bone marrow endothelial progenitor cells (BM EPCs) can improve hematopoiesis and megakaryocytopoiesis, and regulate thrombopoiesis (25). Correcting endothelial dysfunction added a promising tool to the treatment armory of corticosteroid-resistant ITP (26). Regarding the mechanism of IVIG treatment for ITP, interleukin (IL)-11 serves as a stimulating factor to increase platelets and other haemostasis factors (VWF and FVIII), which indicates that recombinant IL-11 (rIL-11) may be a promising substitute for IVIG (27). More comprehensive and detailed pathogenesis can be referred to the reviews by Semple et al. (28) and Zufferey et al. (29). With the discovery of these mechanisms, an increasing number of novel therapeutic approaches are on the horizon. The results of clinical trials of new treatments are summarized in **Table 1**. The drugs in clinical trials are summarized in **Table 2**. Mechanisms of ITP medications are illustrated in **Figure 1**.

**Table 1:** Emerging therapies in refractory ITP.

**Intervention** **/Treatment**	**Mechanism**	**Phase**	**Condition/** **Disease**	**N**	**Major results**	**Adverse events**		**Identifier**	**Ref**
Fostamatinib	Syk inh	2	Chronic refractory ITP	16	a. R: 75% b. SR: 50%	a. 3 patients ended the study for toxicity b. GI toxicity was most common		NCT00706342	Podolanczuk 2008 (30)
Fostamatinib	Syk inh	3	Persistent/ chronic ITP	150	a. Stable response: 18% b. OR: 43% c. Median time to response: 15 d d. Response within 8 w: 83%	Diarrhea 31% Hypertension 28% Nausea 19% Dizziness 11% ALT increase 11%		NCT02076399 NCT02076412	Bussel2018 (31)
Rilzabrutinib (PRN1008)	BTK inh	1/2	Relapsed ITP	60	a. R: 40%	Grade 1 or 2 and transient.		NCT03395210	Kuter2022 (32)
Decitabine	Demethylation	2	Refractory ITP	45	a. CR: 17.78% b. PR: 33.33% c. SR: 44.44%	AE: 28.89%		NCT01568333	Zhou2019 (33)
Oseltamivir	Sialidase inh	2	Newly diagnosed ITP	96	a. R dex+ose vs. dex 86% vs. 66% b. SR dex+ose vs. dex 53% vs. 30%	Fatigue 12% vs. 17% GI reactions 19% vs. 6% Insomnia 16% vs. 9% Anxiety 12% vs. 6%		NCT01965626	Sun2021 (34)
Rozanolixizumab	FcRn-targeting therapeutic	2	ITP	66	R: 50%		Headache 22.7% Vomiting 7.6% Diarrhea 6.1%	NCT02718716	Robak2020 (35)
Efgartigimod	FcRn-targeting therapeutic	2	ITP	38	a. Platelet count ≥50 × 10^9^/L on at least two occasions: 46.2% b. Platelet count ≥50 × 10^9^/L for a cumulative duration of more than 10 d: 38.5%		No dose‐related safety observations	NCT03102593	Newland2019 (36)
Sirolimus	mTOR inh	1/2	Chronic and/or refractory autoimmune cytopenia	30	a. 6% patients with multilineage cytopenias secondary to CVID, ES, or SLE achieved CR b. All children with ALPS achieved CR		Mucositis 33.33%	NCT00392951	Bride2016 (37)
PRTX-100	SpA	1/2	Persistent/ chronic ITP	6	Platelet count elevation was observed on Day 3 and remained elevated for 2-3 w		Acceptable safety profile	NCT02566603 NCT02401061	Bussel2016 (38)
Rozrolimupab (Sym001)	Recombinant anti-D monoclonal antibodies	2	RhD^+^ ITP	61	a. R after 72 hours: 62% b. R within 24 hours: 23% c. Median duration: 14d		Headache 20%	NCT00718692	Robak2012 (39)

N, patient number; inh, inhibitor; R, response; SR, sustained response; GI, gastrointestinal; OR, overall response; d, day; w, week; ALT, alanine transaminase; CR, complete response; PR, partial response; AE, adverse event; dex, dexamethasone; ose, oseltamivir; CVID, common variable immunodeficiency; ES, Evans syndrome; SLE, systemic lupus erythematosus; ALPS, autoimmune lymphoproliferative syndrome; SpA, staphylococcus protein A.

**Table 2:** Ongoing clinical trials of new drugs for refractory ITP.

**Intervention** **/Treatment**	**Mechanism**	**Phase**	**Condition/** **Disease**	**Route of administration**	**Identifier**
SKI-O-703	Syk inh	2	Persistent and chronic ITP	Oral	NCT04056195
HMPL-523	Syk inh	1	ITP	Oral	NCT03951623
Orelabrutinib	BTK inh	2	Refractory ITP	Oral	NCT05020288
Rilzabrutinib (PRN1008)	BTK inh	3	Persistent or chronic ITP	Oral	NCT04562766
Bortezomib	Proteasome inh	2	ITP	IV	NCT03013114
Bortezomib+R vs. RTX	Proteasome inh Anti-CD20	3	Newly diagnosed ITP	SC vs. IV	NCT03443570
Decitabine	Demethylation	3	Newly diagnosed ITP	IVGTT	NCT03252457
Oseltamivir	Sialidase inh	3	ITP	Oral	NCT03520049
Efgartigimod	FcRn-targeting therapeutic	3	ITP	IV	NCT04188379 NCT04225156
Efgartigimod	FcRn-targeting therapeutic	3	ITP	SC	NCT04812925 NCT04687072
Rozanolixizumab	FcRn-targeting therapeutic	3	Persistent or chronic ITP	SC	NCT04596995 NCT04224688 NCT04200456
HBM9161	FcRn-targeting therapeutic	2/3	ITP	IV	NCT04428255
GL-2045	FcR–targeting biologics	1	ITP	SC IV	NCT03275740
Atorvastatin	Improve endothelial function	2/3	Newly diagnosed ITP	Oral	NCT03692754
Chidamide	Histone deacetylase inh	2	Refractory ITP	Oral	NCT03838354
UC-MSCs	Improving immune tolerance	N/A	Refractory ITP	–	NCT04014166

inh, inhibitor; IV, intravenous injection; RTX, rituximab; SC, subcutaneous injection; IVGTT, intravenously guttae; UC-MSCs, human umbilical cord-derived mesenchymal stem cells; N/A, not available.

## 3 Therapeutics targeting macrophages

### 3.1 Spleen tyrosine kinase inhibitor

Spleen tyrosine kinase (Syk), a protein tyrosine kinase, is regarded as a pivotal regulator in signaling pathways implicated in ITP development and progression. When anti-platelet IgG antibodies bind to surface antigens, platelets become vulnerable to Syk-dependent FcγR-mediated phagocytosis by macrophages (40). Syk inhibitor ameliorates platelet destruction in ITP patients by disrupting this process.

Fostamatinib is the first approved Syk inhibitor for chronic ITP patients who failed prior therapies. A phase 2 clinical trial (NCT00706342) revealed that the substantial and sustained clinical response rates in the fostamatinib group were 75% and 50%, respectively (30). Most of these patients failed to respond to rituximab, splenectomy or TPO-RA. Subsequently, in the phase 3 FIT1 and FIT2 trials (NCT02076399 and NCT02076412), 18% of patients in the fostamatinib group achieved stable response and 43% achieved overall response (31). Moreover, fostamatinib administered as a second-line treatment rather than a third- or later-line treatment for ITP has proven better effectiveness (41). Diarrhea, hypertension, nausea, dizziness, and a rise in ALT were the most common adverse events (AEs), which were mild to moderate and resolved spontaneously or with medical intervention (31). Although patients with ITP have bleeding risk, thromboembolic events (TEEs) still warrant caution as previous study has found ITP patients at higher risk of thromboembolism than general population (42). In the latest FIT3 open-label extension study with 146 patients, the incidence of TEEs with fostamatinib for up to 5 years was 0.7% (43), which is lower than TPO-RA. The reason could be that fostamatinib increases platelet count primarily by inhibiting macrophage phagocytosis in ITP rather than stimulating the production of platelets. Meanwhile, the decreased thrombosis rate in ITP patients treated with fostamatinib does not generate an increased risk of bleeding. Fostamatinib is orally administrated and has no dietary restrictions, which promotes patient compliance. The recommended initial dosage is 100 mg bid, and then the dosage may escalate to 150 mg bid if platelet count remains <50×10^9^/L after 4 weeks (44). Whether higher doses produce higher response rates remains unanswered. If the platelet count does not ascend to a hemostatic level, the drug should be withheld after 12 weeks. Collectively, Syk inhibitors play a potentially specific role in difficult-to-treat, long-term, severe and refractory ITP.

### 3.2 Bruton’s tyrosine kinase inhibitor

Bruton’s tyrosine kinase (BTK) belongs to the TEC kinase family, and is ubiquitously expressed in B lymphocytes, myeloid cells, neutrophils, mast cells and osteoclasts (45). BTK mediates the development, proliferation, trafficking, apoptosis, and antibody production of B cells. It also mediates the activation of macrophages and regulates phagocytosis. BTK abnormality can induce autoimmune diseases. BTK inhibitor is of high clinical value in B-cell malignancies and has potential clinical applications in autoimmune diseases (46, 47). Rilzabrutinib (PRN1008), a reversible BTK inhibitor, has exhibited well-tolerated safety in healthy volunteers (48). In a clinical trial of relapsed ITP patients treated with rilzabrutinib (PRN1008) (NCT03395210), rilzabrutinib led to rapid and consistently reliable clinical activity. 40% patients reached a platelet count of 50×10^9^/L on at least two occasions and the median response time was 11.5 days (32). Treatment-emergent adverse events were mild, primarily affecting the gastrointestinal tract. To further evaluate the effectiveness and toxicity of rilzabrutinib (PRN1008) in refractory ITP, a phase 3 study (NCT04562766) is undergoing. Orelabrutinib is an oral, irreversible and highly selective BTK inhibitor that has recently been applied in mantle cell lymphoma (MCL) and lymphocytic leukemia patients (49). A phase 2 study of orelabrutinib for refractory ITP is in progress (NCT05020288).

### 3.3 PRTX-100

Staphylococcus protein A (SpA), a virulence factor generated by staphylococcus aureus, has immunomodulatory function by binding to the Fc region of IgG, variable region of VH3 encoded antibodies and antigen-binding domain of IgM molecules exposed on the B cells (50–52). In 2014, Bernton et al. proposed that PRTX-100, a SpA preparation, may suppress platelet phagocytosis by macrophages (11). In a preclinical study of ITP, PRTX-100 displayed the same ability to block platelet clearance as IVIG (53), with uncertain mechanisms. In two dose-escalation trials of PRTX-100 for ITP (NCT02566603 and NCT02401061), platelet count increased on day 3 and remained elevated for 2-3 weeks with an acceptable safety profile (38).

### 3.4 Neonatal Fc receptor (FcRn)-targeting therapeutics

The neonatal Fc receptor (FcRn) is mainly responsible for maintaining IgG homeostasis, which involves in the transport, circulation, and clearance of IgG. Inhibiting FcRn offers an innovative therapeutic alternative for ITP by selectively decreasing total serum IgG without affecting IgM or IgA. There is evidence from mouse models that inhibiting FcRn has a potential therapeutic benefit in antibody-mediated autoimmune and alloimmune conditions (54). Investigators found that anti-mouse FcRn monoclonal antibodies ameliorated the reduction in platelet counts and protected freshly generated platelets from degradation (55). Subcutaneous (SC) infusion of rozanolixizumab, a humanized IgG4 anti-FcRn monoclonal antibody, impedes the binding of IgG to FcRn, thereby inhibiting IgG recycling. In a phase 1 study (NCT02220153), rozanolixizumab reduced plasma IgG levels in a dose dependent way. The SC route of administration exhibited a comparatively higher degree of safety and tolerability than the intravenous injection (IV) route (56). In a phase 2 study (NCT02718716) of 66 persistent or chronic primary ITP patients with previous therapies (median, 4), more than half of the patients in the 15 and 20 mg/kg single-dose groups had clinically meaningful platelet responses, lasting 10 to 12 days (35). The long duration of response and the convenient route of administration provides a home-infusion option for patients. Efgartigimod is a monoclonal IgG1 Fc fragment that possesses increased affinity to FcRn (57). In a phase 2 study of ITP patients who did not respond to previous therapies (NCT03102593), efgartigimod caused a rapid reduction in total IgG levels, and 46% of patients in the efgartigimod group reached a platelet count of 50×10^9^/L on at least two occasions, compared to 25% in the placebo group (36). Efgartigimod manifested a favorable tolerability profile with no dose-related side effects (36). Moreover, clinical trials have found that these two FcRn antagonists did not increase the infection rate (35, 36, 56, 58). Compared with IVIG, FcRn antagonist has an improved safety profile, avoidance of exposure to blood products and less toxic side effect (58, 59). Both rozanolixizumab and efgartigimod are in phase 3 trials for further evaluating FcRn-targeting therapeutics as a potential approach in refractory ITP.

### 3.5 Recombinant fragment crystallizable (rFc) multimers

GL-2045 is a fully recombinant Fc multimer in which the human IgG2H sequence is added to the C-terminus of mice IgG2a (60). GL-2045 displayed high affinity for Fc receptors and prevented the circulating immune complexes from binding to human FcγRs in rheumatoid arthritis (RA) patients (61). GL-2045 protected platelets from destruction in a murine ITP model (61). A phase 1 clinical study is underway to investigate the safety and tolerability of GL-2045 (NCT03275740). CSL730 contains three human IgG1 Fc fragments connected in a configuration of “Y” between the hinge of the lower Fc fragment and the C-termini of the upper Fc fragment (62). The efficacy of CSL730 is demonstrated to be 40-fold higher than IVIG in animal models of ITP (62). Hexametric-Fc fusion protein (hexameric-Fc) consisting of human IgG1 and IgG4 Fc domains effectively blocked FcγR in murine models of ITP (63, 64).

### 3.6 Fc/FcγR -targeting therapeutics

Fc/FcγR-targeting therapeutics include recombinant soluble FcγRs and monoclonal antibodies. Soluble FcRs were used to neutralize autoimmune IgG. The FcγRIIIA-specific monoclonal antibody 3G8 found in 1982 (65) showed efficacy in refractory ITP patients. However, the development of such FcγR-specific antibodies has been terminated due to AEs, including vomiting, nausea, and fever (66, 67). Nowadays, a novel monovalent anti-FcγRIII/albumin fusion protein has been generated to alleviate platelet destruction without the side effects caused by the FcγR-specific antibody in the ITP murine model (68).

### 3.7 Recombinant anti-D monoclonal antibodies

Anti-D has been utilized as first-line therapeutic approach for ITP. Anti-D is considered to damage the mononuclear phagocytic system by mediating the contact between the Fc fragment and the low-affinity IgG FcγR on immune cells, resulting in immunosuppression (13). This immune-mediated process clears anti-D-coated erythrocytes, which prevents the reticuloendothelial system from destructing antibody-coated platelets (69). Due to life-threatening adverse events of plasma-derived polyclonal anti-D antibodies, such as acute intravascular hemolysis and disseminated intravascular coagulation (DIC) (70, 71), recombinant anti-D monoclonal antibodies were created. Rozrolimupab (Sym001) is comprised of 25 different recombinant anti-D monoclonal antibodies, inducing specific phagocytosis and ADCC of RhD-expressing erythrocytes (69). As a mixture of recombinant antibodies, rozrolimupab exerts its advantages of adequate supplies and no risk of transmission of infectious diseases or other related pathogens. In the phase 2 dose-escalation study (NCT00718692), 300 μg/kg rozrolimupab triggered rapid onset of effect and favorable duration (39). Headache (20%) and pyrexia (13%) were the most frequent AEs (39). Further studies are required to compare the effectiveness and toxicity between rozrolimupab and plasma-derived anti-D Ig preparations.

### 3.8 Monoclonal IgG antibodies to CD44 (anti-CD44)

CD44 is a receptor for hyaluronic acid. The anti-inflammatory effect of anti-CD44 has been shown to play an important role in animal models of autoimmune diseases. The ameliorative effect of anti-CD44 in ITP is comparable to that of IVIG in mouse model (72). Norris et al. found that anti-CD44 blocked the FcγR IgG binding site and thereby inhibited macrophage phagocytosis in an ITP mouse model (73), indicating that anti-CD44 could be a potentially effective alternative to IVIG.

## 4 Therapeutics targeting platelets/megakaryocytes

### 4.1 Platelet desialylation

There is another platelet clearance pathway that is independent of Fc. In this pathway, anti-GPIbα antibodies induce platelet desialylation and desialylated platelets are recognized by the Ashwell-Morell receptor on hepatocyte surface and subsequently phagocytosed (14). This is distinct from the classical FcγR-mediated macrophage phagocytosis, which explains why anti-GPIbα-mediated ITP is resistant to FcγR pathways-targeted treatments. Overexpressed anti-GPIbα antibodies and increased desialylation were demonstrated to be associated with refractory ITP (74). Our research group established a Plt *Slc35a1–/–* mouse model, which exhibited significantly decreased sialylation in megakaryocytes and platelets. We found defective megakaryocytopoiesis, impaired megakaryocyte maturation in Plt *Slc35a1^–/–^* mice, and excessive platelet clearance by Küpffer cells in the liver of Plt *Slc35a1^–/–^* mice, implying that sialylation is vital for platelet homeostasis (75). In the murine model of anti-GPIbα-mediated ITP, the use of sialidase inhibitors ameliorated thrombocytopenia (14). Oseltamivir has been used extensively in anti-influenza treatment by inhibiting viral neuraminidase. In a phase 2 trial (NCT01965626), 96 ITP patients were treated with dexamethasone in combination with oseltamivir or dexamethasone alone. The initial and sustained response rates in the combination treatment cohort were higher than in the dexamethasone cohort, 86% vs. 60% and 53% vs. 30%, respectively. The most common AEs were fatigue, gastrointestinal reactions, insomnia and anxiety (34). In a prospective study of 35 ITP patients, including 16 multirefractory cases, platelet responses were observed in 66.7% of patients treated with oseltamivir combined with TPO-RA or immunosuppressive drugs, compared with 40% in oseltamivir monotherapy group (74). Oseltamivir combined with other medications may lead to preferred outcomes for refractory ITP.

### 4.2 Platelet autophagy

Autophagy is a catabolic process that contributes to the stability and integrity of cellular and tissue. Autophagy has been proved to occur in platelets and be involved in the regulation of hemostasis and thrombosis (76). Using the ATG7 hematopoietic conditional knockout mouse model, Cao et al. discovered that the aberrant autophagy has inhibitory functions on megakaryopoiesis and thrombopoiesis, ultimately causing dysfunctional platelets (15). The autophagy pathway is relevant to the pathogenesis of ITP (77). Deficient autophagy in ITP is owing to the deletion of autophagy-related genes or the overexpression of mammalian target of rapamycin (mTOR) (78). Sirolimus (SRL), previously known as rapamycin, is an mTOR inhibitor for ameliorating transplant rejection. In a multicenter study, SRL led to a low relapse rate and satisfying tolerance (79). In a prospective clinical trial of SRL in 86 patients with refractory ITP, the overall response rate (ORR) at 6 months was 70% and remained 65% at 12 months without severe adverse events (80). Sirolimus outperforms rituximab in terms of efficacy, safety and affordability (81), which is considered as a promising curative alternative for refractory ITP patients. In addition, steroid-dependent patients achieved CR after being treated with SRL, indicating that low-dose steroids and SRL possessed a synergistic response (80). Researchers propose that SRL improves and re-establishes peripheral tolerance by modulating immune cells in ITP patients, which requires further investigations.

### 4.3 Megakaryocyte DNA methylation

DNA methylation is an epigenetic process to regulate gene expression. S-adenosylhomocysteine (SAH) is the product of cellular methyltransferase reactions, and reflects the degree of methylation. In 2008, it was reported that ITP patients had significantly lower mRNA expression of DNA methyltransferase 3A and 3B (DNMT3A and DNMT3B) in peripheral blood mononuclear cells, and increased plasma SAH concentration when compared with healthy controls, indicating aberrant DNA methylation status in ITP (82). Decitabine (DAC), a demethylating agent, has a promotive effect on cell differentiation and maturation at low doses (83). Low-dose DAC promoted megakaryocyte maturation and platelet production through decreasing promoter methylation of tumor necrosis factor-related apoptosis-inducing ligand (TRAIL) (17). Abnormal expression of TRAIL is involved in the apoptosis of megakaryocytes in ITP (84). According to a phase 2 clinical study in 45 patients with refractory ITP (NCT01568333), 17.78% of patients in the low-dose DAC group were in complete response, 33.33% in partial response, and 44.44% in sustained response by 6 months (33). Duration of response was at least 6 months and most responders had response lasted for more than 12 months. In conclusion, low-dose DAC can effectively mitigate refractory ITP with a long-term response. A phase 3 clinical trial comparing dexamethasone combined DAC against dexamethasone monotherapy for ITP is now underway (NCT01568333).

## 5 Therapeutics targeting T cells

### 5.1 PD-1/PD-L1 signaling pathway

PD-1 negatively regulates T-cell activation in peripheral tissues and the tumor microenvironment. PD-L1, a ligand of PD-1, is widely expressed on activated dendritic cells (DCs), Langerhans cells, islet cells and endothelial cells. PD-1/PD-L1 pathway is an immune checkpoint to avoid overactive immune response, the dysfunction of which could lead to inflammatory responses and autoimmune diseases. Increasing data have indicated the importance of this signaling pathway in autoimmune disease pathogenesis (85–88). sPD-1 can hinder the PD-1/PD-L1 pathways while sPD-L1 plays the reversed role. Atesoglu et al. reported lower blood sPD-1 levels in ITP patients when compared to controls (89). However, Wang et al. observed higher percentages of PD-1^+^CD4^+^T cells and PD-L1^+^DCs as well as increased sPD-1 levels in ITP, implying inhibited PD-1/PD-L1 pathway in ITP (18). Afterward, Wu et al. found that the disequilibrium of immune cells in ITP patients could be restored when PD-1/PD-L1 was stimulated by sPD-L1 (90). In conclusion, the PD-1/PD-L1 pathway contributes to ITP through disrupting immune balance, which may present an innovative therapy for patients with ITP.

### 5.2 DNA methylation (Treg cell and cytotoxic T lymphocyte)

In addition to its involvement in megakaryocyte maturation mentioned above, DAC can re-establish immunological tolerance in ITP by regulating Treg cell and CTLs. In pediatric ITP patients, methylation level of the Foxp3 promoter was higher than controls, which induced deficient Treg cells (91). In preclinical studies, Han et al. analyzed the implication of DAC on T-cell subpopulations in ITP, and observed that low-dose DAC rebalanced Treg cell homeostasis and amplified their immunosuppressive function (20). They also found low-dose DAC reduced CTLs against platelets in ITP by restoring the methylation level of programmed cell death protein 1 in CD8^+^ T cells (21).

### 5.3 CTLA4 histone acetylation

Low-dose histone deacetylase inhibitors have immunomodulatory activity (92). The H3K27 histone acetylation anomaly in *CTLA4* gene caused low-expression of CTLA4 protein, and led to reduced Treg cell quantity and activities, as well as ITP pathogenesis (19). Chidamide, a histone deacetylase inhibitor initially applied in anti-neoplastic treatment, was found to inhibit macrophage phagocytosis and promote Treg cell proliferation by upregulating CTLA4 expression, which ameliorated thrombocytopenia (19). A phase 2 clinical trial of low-dose chidamide in refractory ITP patients has been initiated (NCT03838354).

### 5.4 Mesenchymal stem cells and immunological tolerance

Mesenchymal stem cells (MSCs) are mesodermal-derived adult stem cells with multidirectional differentiation potential and immunomodulatory features (93). The primary sources of MSCs in clinical practice are bone marrow, muscle, umbilical cord blood, and adipose tissue. Bone marrow MSCs (BM-MSCs) of ITP patients have a defective immunosuppressive potential and Treg-inducing capacity compared to controls, which indicated that the immunological dysfunction of MSCs is associated with ITP development (94). Therefore, correcting the MSCs defect could represent an alternative therapeutic option for ITP, particularly for refractory cases. MSCs have been shown to improve immunological tolerance in individuals with refractory ITP by regulating immune cell homeostasis. BM-MSCs increased platelet counts through the generation of suppressive cytokines and the upregulation of Tregs in mice (95). In ITP mice receiving adipose-derived MSCs (ADSCs) transplantation, their thrombocytopenia significantly improved, which was related to decreased level of pro-inflammatory T helper (Th) cytokines (96). Human umbilical cord-derived MSCs (UC-MSCs) are also capable of alleviating refractory ITP (97), with clinical research of UC-MSCs for refractory ITP ongoing (NCT00718692). In a clinical case report of 4 refractory ITP patients, 3 patients were in CR within 12 months and 1 patient was in CR within 24 months without severe AEs during the whole treatment procedure (98). The underlying mechanism of how MSCs affect ITP has not been absolutely elucidated (99, 100).

## 6 Therapeutics targeting B cells

Plasma cells can be divided into short-lived plasma cells, residing primarily in the spleen or lymph nodes and undergoing apoptosis rapidly after antibody synthesis, and LLPCs, which have long-term survival, reside immobilized in the bone marrow and continuously produce antibodies (101, 102). Mainstream therapies like glucocorticoids, IVIG and anti-CD20 aim to suppress B cells or short-lived plasma cells rather than LLPCs. LLPCs were involved in the development of ITP through the continuous secretion of anti-platelet antibodies, particularly in relapsed ITP (24). Bortezomib is a proteasome inhibitor that has been commonly used in clinical practice for multiple myeloma and MCL. It stimulates the apoptosis of both LLPCs and short-plasma cells, thus mitigating thrombocytopenia (24). Bortezomib has been used to treat thrombotic thrombocytopenic purpura (TTP) with effectiveness (103, 104) Some case reports showed the curative effect of bortezomib in ITP (105, 106). While data for bortezomib in refractory ITP are not available, we believe such an approach may open new avenues to surmount therapy resistance of ITP.

The splenic microenvironment caused by B-cell depletion therapy (e.g., rituximab) paradoxically promotes LLPC generation and settlement in the spleen, which explained rituximab failure (107). B-cell activating factor (BAFF) is necessary for the survival of splenic LLPCs in the context of B-cell depletion (108). The combination of anti-CD20 and anti-BAFF contributed to the elimination of splenic plasma cells (108). Belimumab is an anti-BAFF monoclonal antibody which is approved for systemic lupus erythematosus (SLE) (109). A phase 2b study of 15 persistent or chronic ITP patients reported that the combination of rituximab and belimumab led to an 80% overall response rate and a 66.7% CR rate at 12 months, with no severe hypogammaglobulinemia observed (NCT03154385) (110).

## 7 Therapeutics targeting endothelial cells

Platelets and endothelial cells are intimately related. On one hand, platelets produce proangiogenic cytokines and synthesize or transport several key endothelial cell trophogens to maintain vascular integrity. On the other hand, endothelial cells secrete various trophic cytokines to support megakaryocyte development and platelet production (111). BM EPCs have been proved to improve hematopoiesis and megakaryocytopoiesis, and regulate thrombopoiesis (25, 111). Researchers first found endothelial cell alterations in a canine ITP model (112). Subsequently, it was found that corticosteroid-resistant ITP patients had endothelial dysfunction (26). Targeted improvement of endothelial cell function may offer new option for refractory ITP. Atorvastatin is frequently applied to reduce blood lipid and protect blood vessels. Yuan et al. detected that atorvastatin could alleviate thrombocytopenia by improving BM EPC function and quantity through downregulating the p38 MAPK pathway in corticosteroid-resistant ITP patients and partially recover BM EPCs mediated promotion of megakaryopoietic function (113). A pilot cohort study of corticosteroid-resistant ITP patients reported a CR of 23.1% and an overall response rate of 69.2% with no apparent AEs (113). However, no clinical trial results are available on the efficacy of atorvastatin for refractory ITP.

## 8 Combination treatments for refractory ITP

### 8.1 TPO-RA-based combination treatments

A multicenter study published in 2016 by Mahévas et al. reported on a total of 37 patients with refractory ITP who had previously failed to respond to splenectomy, rituximab and TPO-RA. The results showed that 7.14% patients achieved response after immunosuppressant therapy and 70% patients achieved response after the addition of TPO-RA to immunosuppressant therapy with a median response duration of 15 months (5).

In 2020, the combination of mycophenolate mofetil (MMF) or cyclosporine (CsA), TPO-RA and IVIG showed promising results in severe refractory ITP with a response rate of 72.2%. Of the 18 patients who received the combination therapy, 11 developed headache and/or abdominal discomfort. The researchers proposed that oral fostamatinib can inhibit phagocytosis as an alternative to IVIG with a more convenient route of administration (114).

### 8.2 Rituximab-based combination treatments

A phase 2b study of 20 chronic ITP patients who received dexamethasone, CsA and low-dose rituximab reported a response rate of 60% and relapse-free survival rates of 92% and 76% at 12 and 24 months, respectively. This triple regimen has a treatment period of 4 weeks, while 60% of patients achieved long-term remission (≥7 months) without further therapy, which is advantageous in terms of reducing the financial burden on patients (115).

In 2019, Wang et al. retrospectively analyzed the therapeutic effects of combining rituximab with cyclophosphamide (CTX) on 249 refractory ITP patients. 41.77% patients receiving rituximab alone and 15.12% receiving CTX achieved CR, whereas 69.05% patients in the combination group achieved CR. The incidence of AEs was 29.11%, 40.7%, and 14.29%, respectively (116).

In addition, the combination of rituximab and belimumab has been illustrated in “therapeutics targeting B cells” section (110).

### 8.3 The combination of rituximab and TPO agents

One multicenter randomized study compared the combination of rituximab with recombinant human thrombopoietin (rhTPO) and rituximab monotherapy in 115 corticosteroid-resistant or relapsed ITP patients. The ORR were 79.2% and 71.1% in combination group and rituximab monotherapy group, respectively. The CR were 45.4% and 23.7% in combination group and rituximab monotherapy group, respectively. Combining rituximab with rhTPO led to shorter time to response but did not lead to improved long-term response (NCT01525836) (117).

### 8.4 Others

In a phase 2 clinical trial, all-trans retinoic acid (ATRA) plus danazol was compared with danazol in 93 ITP patients who had not undergone splenectomy and were refractory to or relapsed after steroid administration. After 12 months of follow-up, the combination group reached superior sustained response (62% vs. 25%) (NCT01667263) (118).

## 9 Future and perspective

In low-income countries, financial capacity and drug availability play an important role in determining the treatment options for refractory ITP. The medications that are inexpensive and readily available are more feasible options in this scenario. Immunosuppressants, danazol, dapsone are relatively inexpensive, but it is recommended to combine these drugs with TPO-RA (4). A recent study showed that adding CsA to TPO-RA allowed TPO-RA tapering or discontinuation by increasing platelet counts in refractory patients and stabilizing fluctuations (119). This may alleviate the financial burden of patients to some extent. Currently, sirolimus has shown excellent performance in the treatment of refractory ITP and it is relatively affordable, presenting a promising approach for those in economically underdeveloped areas.

ITP is a heterogeneous disease with its pathogenesis not fully understood. More therapeutic options are becoming available for refractory ITP. Among the drugs summarized above, fostamatinib has been approved for ITP. The efficacy of fostamatinib is comparable to that of rituximab, with a lower stable response rate of 18% (31). It may serve as an alternative option for patients who cannot undergo splenectomy or cannot tolerate or have contradictions to rituximab. Sirolimus has superior overall efficacy and safety than rituximab and TPO-RA (80). It may be an option for rescue therapy of refractory ITP and its combination with glucocorticoids may provide enhanced effectiveness (80). FcRn-targeting therapeutics (rozanolixizumab and efgartigimod) have a short onset of action, representing an alternative to IVIG as a salvage treatment but will be not curative (35, 36). Adding oseltamivir to the combination regimen of TPO-RA or dexamethasone may produce an improved outcome (34, 74). Low-dose DAC has the advantage of short onset period, which is suitable for patients with bleeding symptoms or at risk of bleeding (33). Although atorvastatin has been proven to alleviate thrombocytopenia, it will not revolutionize the treatment of refractory ITP. The role of PD-1/PD-L1 signaling pathway in the pathogenesis of ITP is gradually being discovered, which may serve as a hot spot for future investigation. Novel medications are filling the unmet need for treating refractory ITP while more clinical trials are necessary to investigate their efficacy and safety. The current combination therapy protocols are mainly based on rituximab and TPO-RA (5, 110, 114–116). Further studies are demanded to elucidate whether a combination of these novel drugs could trigger improved efficacy in the treatment of refractory ITP.

## Author contributions

YL wrote the initial draft, which was revised and amended by HS, HL, and LZ. All authors contributed to the article and approved the submitted version.

## Conflict of interest

The authors declare that the research was conducted in the absence of any commercial or financial relationships that could be construed as a potential conflict of interest.

## Publisher’s note

All claims expressed in this article are solely those of the authors and do not necessarily represent those of their affiliated organizations, or those of the publisher, the editors and the reviewers. Any product that may be evaluated in this article, or claim that may be made by its manufacturer, is not guaranteed or endorsed by the publisher.
